# PRM-based quantitative proteomics analysis of altered HSP abundance in villi and decidua of patients with early missed abortion

**DOI:** 10.1186/s12953-023-00213-w

**Published:** 2023-08-16

**Authors:** Xiao-Fang Chen, Xiao-Qing Chen, Hai-Lian Luo, Li-Na Xia, Shu-Hui Huang, Qi Chen

**Affiliations:** 1 Department of Obstetrics and Gynecology, the Second Affiliated Hospital of Nanchang University, Nanchang, 330000 Jiangxi China; 2 Department of Obstetrics and Gynecology, Jiangxi Maternal and Child Health Hospital, Nanchang, 330000 Jiangxi China; 3 Department of Pathology, Jiangxi Maternal and Child Health Hospital, Nanchang, 330000 Jiangxi China; 4 Key Laboratory of Birth Defect for Prevention and Control of Jiangxi Province, Jiangxi Maternal and Child Health Hospital, Nanchang, 330000 Jiangxi China

**Keywords:** Early missed abortion, Proteomics, HSPs, Pregnancy

## Abstract

**Objective:**

In this study, we aimed to identify differentially expressed heat shock protein (HSP) profiles in the villi and decidua from patients with early missed abortion (EMA).

**Methods:**

By using high-throughput and high-precision parallel reaction monitoring (PRM)-based targeted proteomics techniques, this study examined the abundance of HSPs in the villi and decidua of 10 patients with EMA and 10 controls. Moreover, the abundance of 3 HSPs in the villi of another 22 patients with EMA and 22 controls was verified with Western blotting and immunohistochemistry (IHC).

**Results:**

There were potential differences in the abundance of 16 HSPs and 42 polypeptides in human villi and decidua compared with those of the control group. Among them, HSP90AB1, HSPD1 and HSPA13 were downregulated in abundance in villi of patients with EMA, with a statistically significant difference, which was consistent with the verification results of Western blots and IHC.

**Conclusion:**

Using a PRM-based targeted proteomics technique, this study is the first to screen and quantitatively analyze the expression profile of HSPs in the villi and decidua of patients with EMA. The significant downregulation of HSP90AB1, HSPD1 and HSPA13 was found to have a potentially intimate association with the occurrence of EMA. The findings in our study may provide novel potential research targets related to HSPs for the pathogenesis, prevention and treatment of EMA.

**Supplementary Information:**

The online version contains supplementary material available at 10.1186/s12953-023-00213-w.

## Introduction

Early missed abortion (EMA) refers to the phenomenon where an embryo or fetus has died and is retained in the uterine cavity and fails to expel spontaneously during pregnancy ≤ 12 weeks [1]. Although there are many causes of missed abortion, such as maternal endocrine dysfunction, immune dysfunction, reproductive organ anatomical abnormalities and diseases, systemic diseases and infections, chromosomal abnormalities, etc., the etiologies of more than half of cases of missed abortions remain unknown. Therefore, studies on the pathogenesis of missed abortions can pave the way for new research into the prevention and treatment of EMA.

As molecular chaperones, HSPs, which have been widely found at the maternal–embryonic interface, play an important role in protein folding/unfolding, cell cycle regulation and cellular protection [2, 3]. Recently, according to systematic gene symbols, *Kampinga* et al*.*, divided the human HSP family into the following major classes: HSPA (HSP70), HSPC (HSP90), HSPD/E (HSP60/HSP10) and CCT (TRiC), HSPH (HSP110), DNAJ (HSP40) and HSPB (small HSP) [4]. Accumulating evidence suggests that HSPs promote decidualization, implantation and placentation, with dysregulated expression resulting in implantation failure, pregnancy loss and other maternal–fetal complications [3]. For example, HSP27 promotes decidualization [5], HSP105 facilitates placental implantation [6], and altered abundance of Hsp70 may lead to the occurrence of abortion [7–9]. Therefore, we believe that systematic evaluation of HSP abundance in EMA may lead to new molecular targets.

In our study, a PRM-based targeted proteomics technique was used to detect the differential abundance of HSPs at the proteomic level. This method successfully identified the differential abundance of HSPs during malignant melanoma metastasis and revealed that DNAJB4 is a suppressor of melanoma metastasis [10]. Moreover, it has been demonstrated that altered abundance of HSP70, HSP40, and HSPB8 may be associated with radio-resistance development in breast cancer [11]. In this paper, a quantitative analysis was conducted for 42 peptides of 16 target proteins in villi and decidua from 10 patients with EMA and 10 healthy controls to find new molecular targets.

## Materials and methods

### Patients and controls

A total of 32 pregnant women who underwent complete curettage of the uterine cavity in Jiangxi Maternal and Child Health Hospital from January 2020 to December 2020 were enrolled in the case group, and 32 healthy women who voluntarily requested induced abortion due to unintended pregnancy during the corresponding gestational period were enrolled in the control group. In the diagnosis of missed abortion, transvaginal ultrasonography is primarily relied upon to detect empty gestational sacs or embryos/fetuses without cardiovascular beats. All included pregnant women had been screened for fetal chromosomal abnormalities, endocrine diseases, anatomical abnormalities, infections, immune diseases, trauma and medical diseases. All participants were also within 6–10 weeks of gestation and had no history of adverse pregnancy. The embryonic villous tissue and maternal decidual tissue were collected respectively during vacuum aspiration under intravenous anesthesia. All samples were immediately processed in liquid nitrogen and then stored in a -80 °C refrigerator. Ten villus and decidual samples in the EMA group and the control group were selected for PRM-based targeted proteomic analysis (see Table 1 for clinical baseline characteristics of the two groups). Based on the PRM results, Western blotting and immunohistochemistry were performed on another 22 villus samples from the EMA group and the control group. Compared with those of the control group, the clinical parameters of another 22 cases also showed no significant differences in maternal age, BMI, pregnancy duration, gravidity, parity and induced abortion. This study was supported by the Ethics Committee of Jiangxi Maternal and Child Health Hospital. All the studies provided informed consent.

**Table 1:** Comparison of clinical parameters of 10 patients with EMA and 10 controls that underwent HSP expression profiling

	EMA group(*n* = 10)	Control group(*n* = 10)	*P* value
Maternal age(years)	29.60 ± 4.33	30.20 ± 3.05	0.72
BMI (kg/m2)	21.46 ± 1.04	21.09 ± 0.85	0.39
Pregnancy duration(day)	56.50 ± 9.08	57.00 ± 8.71	0.90
Gravidity	1.70 ± 0.67	2.00 ± 0.82	0.38
Parity	1.30 ± 0.67	1.60 ± 0.52	0.27
Induced abortion	0.30 ± 0.48	0.40 ± 0.51	0.66

*BMI* Body mass index

### Protein extraction and enzymatic hydrolysis

After an appropriate amount of SDT lysis solution was added, the sample was transferred to a 2 ml centrifuge tube preloaded with arenaceous quartz and a 1/4-inch ceramic bead MP 6540–424. Homogenization processing was performed using an MP homogenizer (24 × 2, 6.0 M/S, 60 s, twice). In the subsequent steps, the samples underwent an ultrasonic treatment (180 W for 10 s with intervals of 10 s for a total of ten cycles) and were boiled in a water bath for ten minutes. The supernatant was collected by centrifugation at 12,000 × g for 30 min and filtered through a 0.22 µm membrane to obtain the filtrate. Protein quantification was performed using the bicinchoninic acid (BCA) method, and 20 μg of protein from each sample was used for SDS‒PAGE electrophoresis. The areas of degradation were observed as distinct bands against a blue-stained background after Coomassie Brilliant Blue R-250 protein staining. The sample was subpackaged and stored at -80 °C for preservation.

Following enzymatic hydrolysis with FASP, the resulting peptide was subjected to C18 cartridge desalting. The lyophilized peptides were then reconstituted in a 40 µl solution of 0.1% formic acid and quantified using an enzyme-labeled assay.

### Mass spectrometry analysis

A peptide mixture from the sample was prepared, and 1 μg was subjected to chromatographic separation using an HPLC system. Buffer solution A consisted of a 0.1% formic acid aqueous solution, while solution B comprised a 0.1% formic acid acetonitrile aqueous solution (84% acetonitrile). The column was equilibrated with 95% solvent A. The samples were separated via a gradient chromatography column. After high-performance liquid chromatography was conducted, a Q-Exactive HF mass spectrometer (Thermo Scientific) was utilized to perform qualitative analysis via mass spectrometry. The analysis duration was 60 min, and the positive ion detection method was utilized. The scanning range of the parent ion was set at 300–1800 m/z. The primary mass spectrometry resolution reached 60,000 atm/z200. The AGC target was set to 3e6, and the primary maximum IT was limited to 50 ms. The collection of peptides' secondary mass spectrometry was conducted using the following methods: 20 MS2 scans were triggered after each full scan, with a resolution of 15,000 at m/z200 and an AGC target of 1e5. The maximum IT for secondary mass spectrometry was set to 50 ms. HCD was used as the MS2 activation type, with an isolation window of 1.6Th and normalized collision energy set to 27.

The original mass spectrum data were obtained using Proteome Discoverer v.2.2, with database search parameters set to trypsin/P enzyme and 0 missed cleavages. Peptides scoring above 40 were considered reliable, and 1–3 unique peptides were selected for each protein.

Based on the results of protein qualitative analysis, trusted peptides suitable for PRM analysis were imported into Xcalibur mass spectrometry software for PRM method setting. The peptide mixture of 10 µg was subjected to quantitative analysis using both "full scan" and "PRM" modes. The chromatographic separation and full scanning conditions were consistent with those described above. Subsequently, the mass spectrum data were analyzed utilizing Skyline software to determine the availability of selected peptides based on their repeatability and stability.

### PRM detection analysis

The peptide information suitable for PRM analysis mentioned above was imported into Xcalibur software to set up the PRM method. For detection, approximately 1 µg of peptide was taken from each sample, and 20 fmol standard peptide (PRTC: ELGQSGVDTYLQTK) was added. LC-PRM/MS was used with the aforementioned PRM method to detect the target protein in each sample. The original PRM files were analyzed using Skyline 3.5.0, and after a correction was performed for the internal standard peptide signal, the expression level of the target protein in each sample was determined.

### Western blotting validation

Twenty micrograms of the extracted villus protein sample was separated by SDS‒PAGE, transferred to a polyvinylidene fluoride (PVDF) membrane and sealed with 5% defatted milk powder for 1 h. The membrane was incubated with diluted primary antibodies overnight at 4 °C. The membrane was incubated with secondary antibodies for 1 h. The horseradish peroxidase (HRP) signal was detected using hypersensitive enhanced chemical luminescence (ECL) chemical reagent. The target protein bands were analyzed in ImageJ.

Human HSP90AB1 antibody (Boster, Wuhan, China, no. BM4191, 1:2000 dilution), human HSPD1 antibody (Boster, Wuhan, China, no. M01280-3, 1:2000 dilution) and human HSPA13 antibody (Proteintech, Wuhan, China, no. 12667–2-AP, 1:2000 dilution) were used as the primary antibodies for Western blot analysis. The membranes were also incubated with anti-GAPDH antibody (ZSGB-Bio, no. TA-08, 1:10,000 dilution) to verify equal protein loading.

### Immunohistochemistry validation

The paraffin-embedded villus tissue sections were dewaxed and dehydrated as per routine practice. Citric acid antigen repair buffer was used for antigen repair. For blocking of endogenous peroxidase, the slices were incubated in 3% hydrogen peroxide (H2O2) at room temperature for 25 min. The tissue sections were sealed with 3% BSA at room temperature for 30 min and then incubated with 50 μl of diluted primary antibodies overnight at 4 °C. The primary antibody was anti-HSP90AB1 (Boster, Wuhan, China, no. BM4191, 1:200 dilution), anti-HSPD1 (Boster, Wuhan, China, no. M012803, 1:200 dilution) and anti-HSPA13 (Proteintech, Wuhan, China, no. 12667–2-AP, 1:200 dilution). The slices were incubated with the secondary antibodies of the corresponding species of the primary antibody at room temperature for 50 min. Finally, fresh DAB display solution was added to the slices, the color development time was controlled under the microscope, and the positive color was brownish yellow. All sections were analyzed through a modified H-score scoring system [(percentage of weak intensity area × 1) + (percentage of moderate intensity area × 2) + (percentage of strong intensity area × 3)] by two pathologists blinded to the clinical and molecular data. The H-score is a value between 0 and 300, and the larger the value is, the stronger the comprehensive positive intensity [12].

### Statistical analysis

SPSS 20.0 (SPSS, Inc., Chicago, IL, USA) software was used for statistical analysis, and all data are expressed as the mean ± standard deviation (SD). Student's t test was used for comparison of quantitative data between the two groups, and *P* < 0.05 was considered statistically significant. Enumeration data is chi-square test. All experiments were repeated three times.

## Results

### Differentially expressed protein identification

**Fig. 1:**
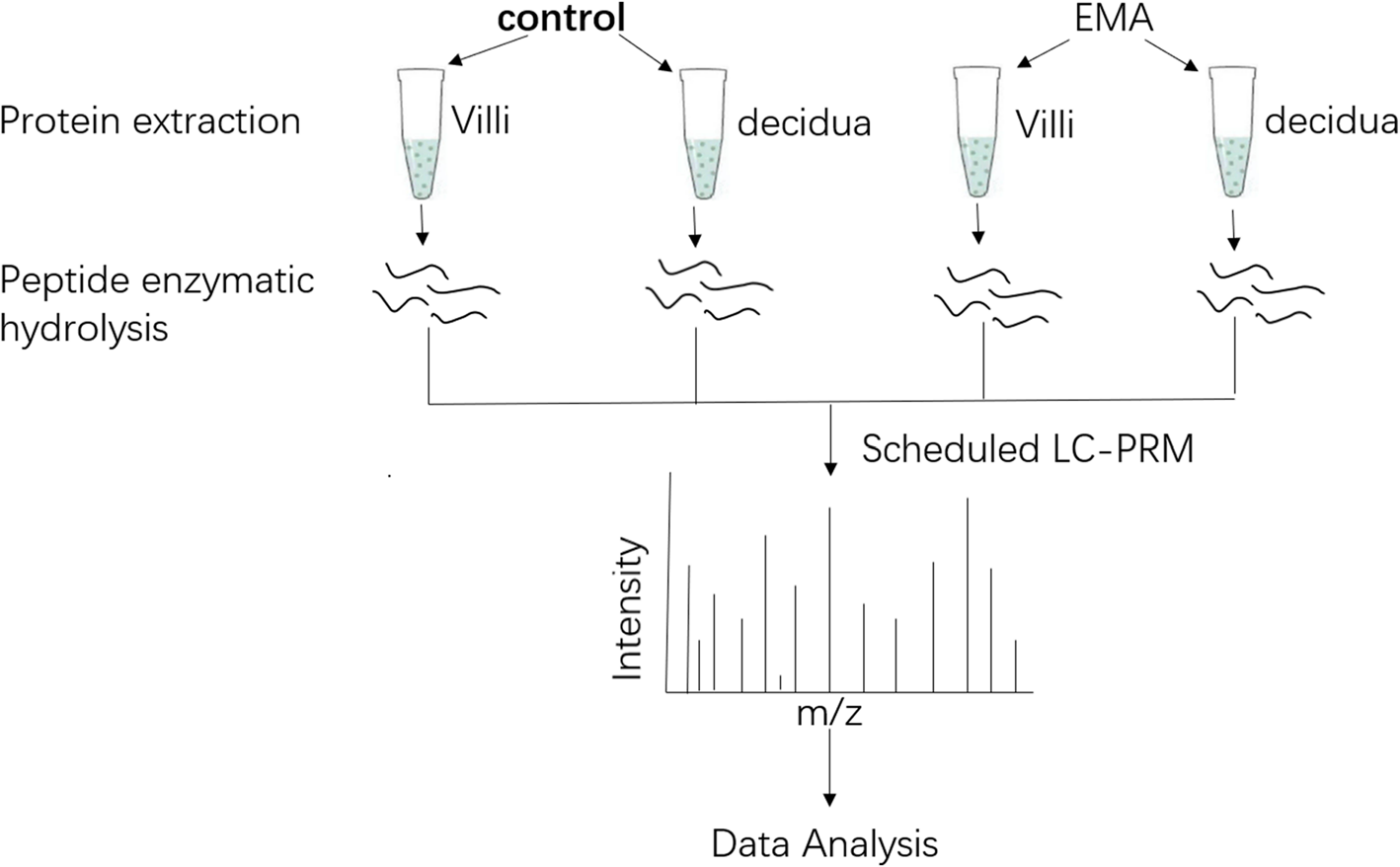
Workflow of preordered LC-PRM analysis for quantitative evaluation of heat shock proteome in villi and decidua of 10 patients with EMA and 10 Controls. All experiments were performed in triplicate

Using PRM-based targeted proteomics, we successfully identified the differentially abundant HSPs in villi and decidua of 10 patients with EMA and 10 controls (Fig. 1). A total of 16 differentially expressed HSPs with 42 polypeptides in total were quantified (Tables 2, 3, 4 and 5). Among them, the abundances of HSP90AB1, HSPD1 and HSPA13 in the villi of the EMA group were decreased compared with those in the control group, with statistically significant differences (all *P* < 0.05); however, these HSPs were not significantly changed in decidual tissues (*P* > 0.05).

**Table 2:** Quantitative and statistical analysis of target peptide in decidua of 10 patients with EMA and 10 controls identified using PRM-based targeted proteomics

No	Peptide sequence	Protein name	Control	EMA	Fold change	*P*-Value
1	IINEPTAAAIAYGLDR	P0DMV8(HSPA1A)	0.82	1.35	1.65	0.41
2	NQVALNPQNTVFDAK	P0DMV8	2.17	1.78	0.82	0.34
3	ATAGDTHLGGEDFDNR	P0DMV8	1.08	1.07	0.99	0.97
4	QSKPVTTPEEIAQVATISANGDK	P10809(HSPD1)	0.20	0.16	0.80	0.40
5	IQEIIEQLDVTTSEYEK	P10809	0.43	0.43	0.99	0.98
6	ISSIQSIVPALEIANAHR	P10809	1.72	0.84	0.49	0.22
7	NPDDITQEEYGEFYK	P08238(HSP90AB1)	1.84	1.42	0.77	0.14
8	HLEINPDHPIVETLR	P08238	0.71	0.47	0.65	0.10
9	EQVANSAFVER	P08238	2.38	2.10	0.88	0.62
10	GGEIQPVSVK	P61604(HSPE1)	2.53	1.98	0.78	0.35
11	VLLPEYGGTK	P61604	4.13	3.39	0.82	0.38
12	VLQATVVAVGSGSK	P61604	2.78	2.25	0.81	0.41
13	LEDTENWLYEDGEDQPK	P34932(HSPA4)	0.24	0.20	0.86	0.39
14	AGGIETIANEYSDR	P34932	0.51	0.47	0.92	0.65
15	KPVVDCVVSVPCFYTDAER	P34932	0.17	0.14	0.80	0.33
16	AFSDPFVEAEK	P34932	0.38	0.31	0.82	0.25
17	SQIFSTASDNQPTVTIK	P11021(HSPA5)	4.73	4.38	0.93	0.70
18	NQLTSNPENTVFDAK	P11021	5.93	4.84	0.82	0.31
19	DNHLLGTFDLTGIPPAPR	P11021	1.23	1.07	0.87	0.58
20	ILVPIQQVLK	P48723(HSPA13)	0.12	0.15	1.30	0.54
21	GQIQEIVLVGGSTR	P54652(HSPA2)	1.59	1.55	0.97	0.85
22	EIAEAYLGGK	P54652	1.44	1.29	0.90	0.63
23	VHSAVITVPAYFNDSQR	P54652	0.57	0.52	0.90	0.65
24	QAVTNPNNTFYATK	P38646(HSPA9)	0.60	0.57	0.95	0.85
25	QAASSLQQASLK	P38646	0.40	0.35	0.87	0.57
26	ASNGDAWVEAHGK	P38646	0.23	0.20	0.88	0.61
27	GNVVPSPLPTR	Q9Y2V2(CARHSP1)	0.92	0.89	0.97	0.87
28	QLSSGVSEIR	P04792(HSPB1)	6.62	7.43	1.12	0.61
29	VSLDVNHFAPDELTVK	P04792	3.48	3.83	1.10	0.68
30	LFDQAFGLPR	P04792	7.66	10.52	1.37	0.55
31	SIEYSPQLEDAGSR	P98160(HSPG2)	1.27	1.62	1.28	0.46
32	SPVISIDPPSSTVQQGQDASFK	P98160	0.25	0.32	1.28	0.52
33	YELGSGLAVLR	P98160	1.27	2.02	1.60	0.32
34	ASAPLPGLSAPGR	O14558(HSPB6)	5.69	6.85	1.20	0.39
35	HFSPEEIAVK	O14558	2.28	2.37	1.04	0.86
36	FQSSHHPTDITSLDQYVER	P14625(HSP90B1)	5.54	5.32	0.96	0.81
37	DISTNYYASQK	P14625	3.01	3.01	1.00	1.00
38	LGVIEDHSNR	P14625	3.12	3.22	1.03	0.92
39	NQQITHANNTVSNFK	Q92598(HSPH1)	0.01	0.01	0.96	0.85
40	ELISNSSDALDK	P07900(HSP90AA1)	1.98	1.55	0.79	0.28
41	DQVANSAFVER	P07900	2.58	1.92	0.75	0.21
42	NPDDITNEEYGEFYK	P07900	1.67	1.20	0.72	0.10

**Table 3:** Quantitative and statistical analysis of target peptide in villi of 10 patients with EMA and 10 controls identified using PRM-based targeted proteomics

No	Peptide sequence	Protein name	Control	EMA	Fold change	*P*-value
1	NPDDITQEEYGEFYK	P08238(HSP90AB1)	1.87	1.04	0.56	0.00
2	HLEINPDHPIVETLR	P08238	0.35	0.33	0.92	0.65
3	EQVANSAFVER	P08238	3.51	1.95	0.56	0.00
4	QSKPVTTPEEIAQVATISANGDK	P10809(HSPD1)	0.36	0.27	0.73	0.17
5	IQEIIEQLDVTTSEYEK	P10809	0.89	0.28	0.31	0.02
6	ISSIQSIVPALEIANAHR	P10809	7.18	3.13	0.44	0.07
7	ILVPIQQVLK	P48723(HSPA13)	0.18	0.08	0.43	0.05
8	AGGIETIANEYSDR	P34932(HSPA4)	0.57	0.42	0.73	0.21
9	KPVVDCVVSVPCFYTDAER	P34932	0.15	0.13	0.86	0.42
10	AFSDPFVEAEK	P34932	0.49	0.33	0.67	0.08
11	ELISNSSDALDK	P07900(HSP90AA1)	2.15	1.85	0.86	0.37
12	DQVANSAFVER	P07900	3.17	2.35	0.74	0.16
13	NPDDITNEEYGEFYK	P07900	1.71	1.12	0.66	0.10
14	GQIQEIVLVGGSTR	P54652(HSPA2)	2.01	1.31	0.65	0.23
15	EIAEAYLGGK	P54652	0.69	0.70	1.01	0.96
16	VHSAVITVPAYFNDSQR	P54652	0.36	0.30	0.83	0.35
17	SQIFSTASDNQPTVTIK	P11021(HSPA5)	18.81	12.34	0.66	0.16
18	NQLTSNPENTVFDAK	P11021	20.20	14.75	0.73	0.28
19	DNHLLGTFDLTGIPPAPR	P11021	3.89	3.85	0.99	0.98
20	QAVTNPNNTFYATK	P38646(HSPA9)	1.18	0.89	0.75	0.14
21	QAASSLQQASLK	P38646	0.78	0.63	0.81	0.36
22	ASNGDAWVEAHGK	P38646	0.44	0.38	0.85	0.45
23	GGEIQPVSVK	P61604(HSPE1)	5.71	5.31	0.93	0.73
24	VLLPEYGGTK	P61604	9.07	7.07	0.78	0.16
25	VLQATVVAVGSGSK	P61604	5.58	4.99	0.89	0.59
26	NQQITHANNTVSNFK	Q92598(HSPH1)	0.01	0.01	0.83	0.40
27	FQSSHHPTDITSLDQYVER	P14625(HSP90B1)	12.77	13.05	1.02	0.93
28	DISTNYYASQK	P14625	8.57	6.42	0.75	0.17
29	LGVIEDHSNR	P14625	8.93	9.09	1.02	0.93
30	FQSSHHPTDITSLDQYVER	P14625	12.77	13.05	1.02	0.93
31	IINEPTAAAIAYGLDR	P0DMV8(HSPA1A)	1.26	2.19	1.74	0.16
32	NQVALNPQNTVFDAK	P0DMV8	1.13	1.22	1.09	0.64
33	ATAGDTHLGGEDFDNR	P0DMV8	0.68	1.05	1.54	0.03
34	SIEYSPQLEDAGSR	P98160(HSPG2)	1.43	1.71	1.20	0.45
35	SPVISIDPPSSTVQQGQDASFK	P98160	0.24	0.30	1.29	0.44
36	YELGSGLAVLR	P98160	1.08	1.53	1.42	0.27
37	QLSSGVSEIR	P04792(HSPB1)	6.92	7.43	1.07	0.82
38	VSLDVNHFAPDELTVK	P04792	2.05	1.93	0.95	0.83
39	LFDQAFGLPR	P04792	5.36	9.11	1.70	0.11
40	GNVVPSPLPTR	Q9Y2V2(CARHSP1)	0.43	0.56	1.28	0.36
41	ASAPLPGLSAPGR	O14558(HSPB6)	1.39	1.16	0.84	0.48
42	HFSPEEIAVK	O14558	0.35	0.60	1.71	0.11

**Table 4:** Differentially expressed HSPs proteins in decidua of 10 patients with EMA and 10 controls identified using PRM-based targeted proteomics

No	Protein name	Gene name	Uniprot ID	Fold change	*P*-Value
**Down-regulated HSPs in decidua tissue with EMA**					
1	Heat shock protein HSP 90-alpha	HSP90AA1	P07900	0.75	0.15
2	60kDa heat shock protein, mitochondrial	HSPD1	P10809	0.61	0.18
3	Heat shock protein HSP 90-beta	HSP90AB1	P08238	0. 81	0.30
4	10kDa heat shock protein, mitochondrial	HPSE1	P61604	0.81	0.37
5	Heat shock 70 kDa protein 4	HSPA4	P34932	0.86	0.41
6	Endoplasmic reticulum chaperone BiP	HSPA5	P11021	0.86	0.44
7	Heat shock 70 kDa protein 13	HSPA13	P48723	1.3	0.54
8	Heat shock-related 70 kDa protein 2	HSPA2	P54652	0.93	0.69
9	Stress-70 protein, mitochondrial	HSPA9	P38646	0.91	0.70
10	Calcium-regulated heat-stable protein 1	CARHSP1	Q9Y2V2	0.97	0.87
11	Endoplasmin	HSP90B1	P14625	0.99	0.96
**Up-regulated in decidua tissue with EMA**					
12	Basement membrane-specific heparan sulfate proteoglycan core protein	HSPG2	P98160	1.42	0.35
13	Heat shock protein beta-6	HSPB6	O14558	1.16	0.50
14	Heat shock protein beta-1	HSPB1	P04792	1.23	0.53
15	Heat shock protein 105 kDa	HSPH1	Q92598	0.96	0.85
16	Heat shock 70 kDa protein 1A	HSPA1A	P0DMV8	1.03	0.91

**Table 5:** Differentially expressed HSPs proteins in villi of 10 patients with EMA and 10 controls identified using PRM-based targeted proteomics

No	Protein name	Gene name	Uniprot ID	Fold change	*P*-Value
**Down-regulated HSPs in villi tissue with EMA**					
1	Heat shock protein HSP 90-beta	HSP90AB1	P08238	0.58	0.0008
2	60kDa heat shock protein, mitochondrial	HSPD1	P10809	0.44	0.039
3	Heat shock 70 kDa protein 13	HSPA13	P48723	0.44	0.048
4	Heat shock 70 kDa protein 4	HSPA4	P34932	0.69	0.11
5	Heat shock protein HSP 90-alpha	HSP90AA1	P07900	0.76	0.14
6	Heat shock-related 70 kDa protein 2	HSPA2	P54652	0.74	0.17
7	Endoplasmic reticulum chaperone BiP	HSPA5	P11021	0.72	0.23
8	Stress-70 protein, mitochondrial	HSPA9	P38646	0.79	0.23
9	10kDa heat shock protein, mitochondrial	HPSE1	P61604	0.85	0.37
10	Heat shock protein 105 kDa	HSPH1	Q92598	0.83	0.40
11	Endoplasmin	HSP90B1	P14625	0.94	0.79
**Up-regulated in villi tissue with EMA**					
12	Heat shock 70 kDa protein 1A	HSPA1A	P0DMV8	1.46	0.11
13	Basement membrane-specific heparan sulfate proteoglycan core protein	HSPG2	P98160	1.29	0.32
14	Heat shock protein beta-1	HSPB1	P04792	1.29	0.35
15	Calcium-regulated heat-stable protein 1	CARHSP1	Q9Y2V2	1.28	0.36
16	Heat shock protein beta-6	HSPB6	O14558	1.01	0.95

### Western blotting validation of PRM-based results

**Fig. 2:**
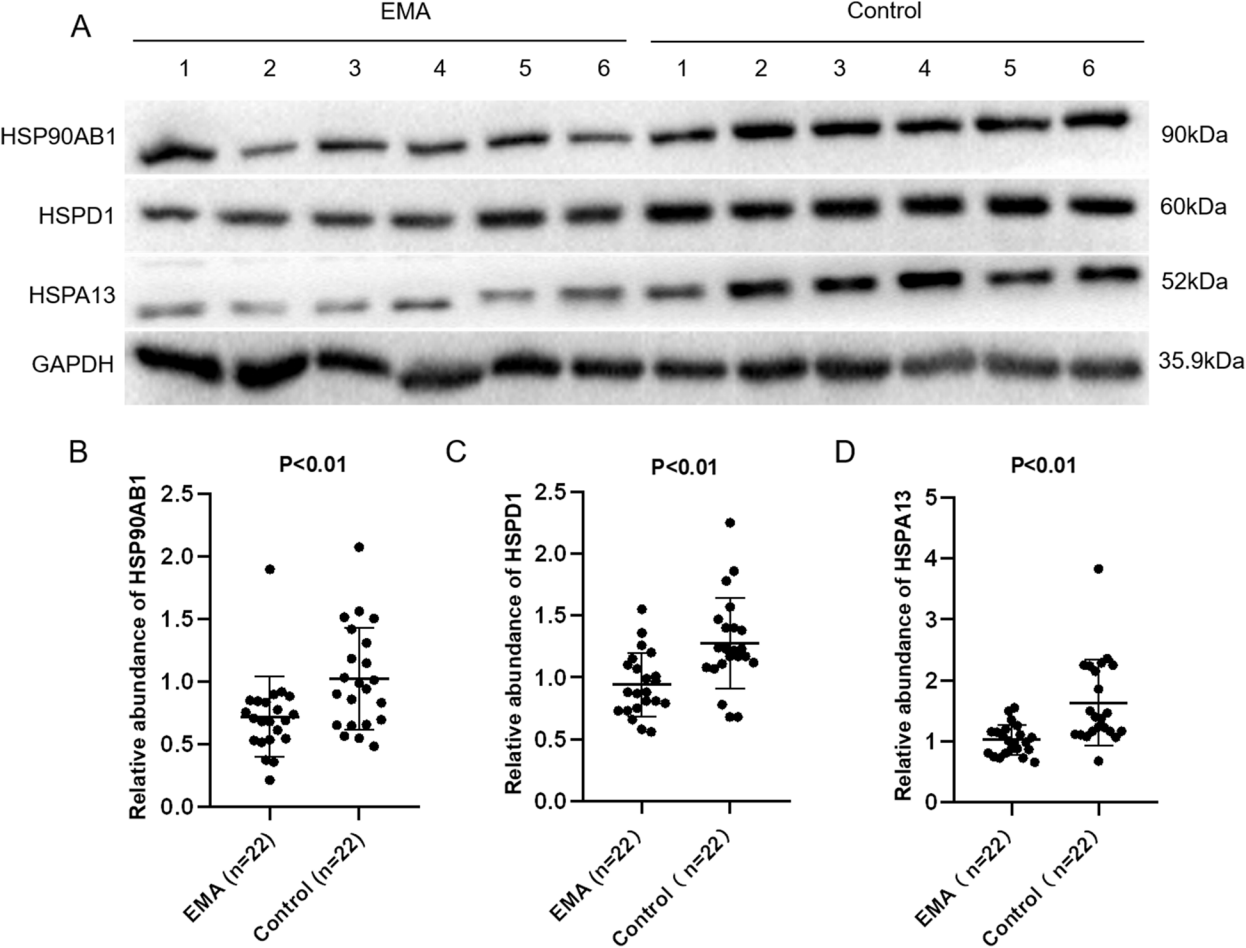
The differential abundance of HSP90AB1, HSPD1 and HSPA13 in villi tissues was verified by Western blot. **A** The representative of Western blot analysis to verify selected differentially expressed proteins HSP90AB1, HSPD1 and HSPA13 in the villi tissue of embryo from EMA (*n* = 6) and control(*n* = 6). **B** The scatter plot of HSP90AB1 abundance in the villi tissue of embryo from EMA (*n* = 22) vs control (*n* = 22) (*P* < 0.01). **C** The scatter plot of HSPD1 abundance in the villi tissue of embryo from EMA (*n* = 22) vs control (*n* = 22) (*P* < 0.01). **D** The scatter plot of HSPA13 abundance in the villi tissue of embryo from EMA (*n* = 22) vs control (*n* = 22) (*P* < 0.01)

The Western blotting results showed that the abundance levels of HSP90AB1, HSPD1, and HSPA13 were decreased in 22 cases of EMA villi, with statistically significant differences compared to the control group (Fig. 2). The above results were consistent with those verified by the PRM-based method.

### Immunohistochemistry validation of PRM-based results

**Fig. 3:**
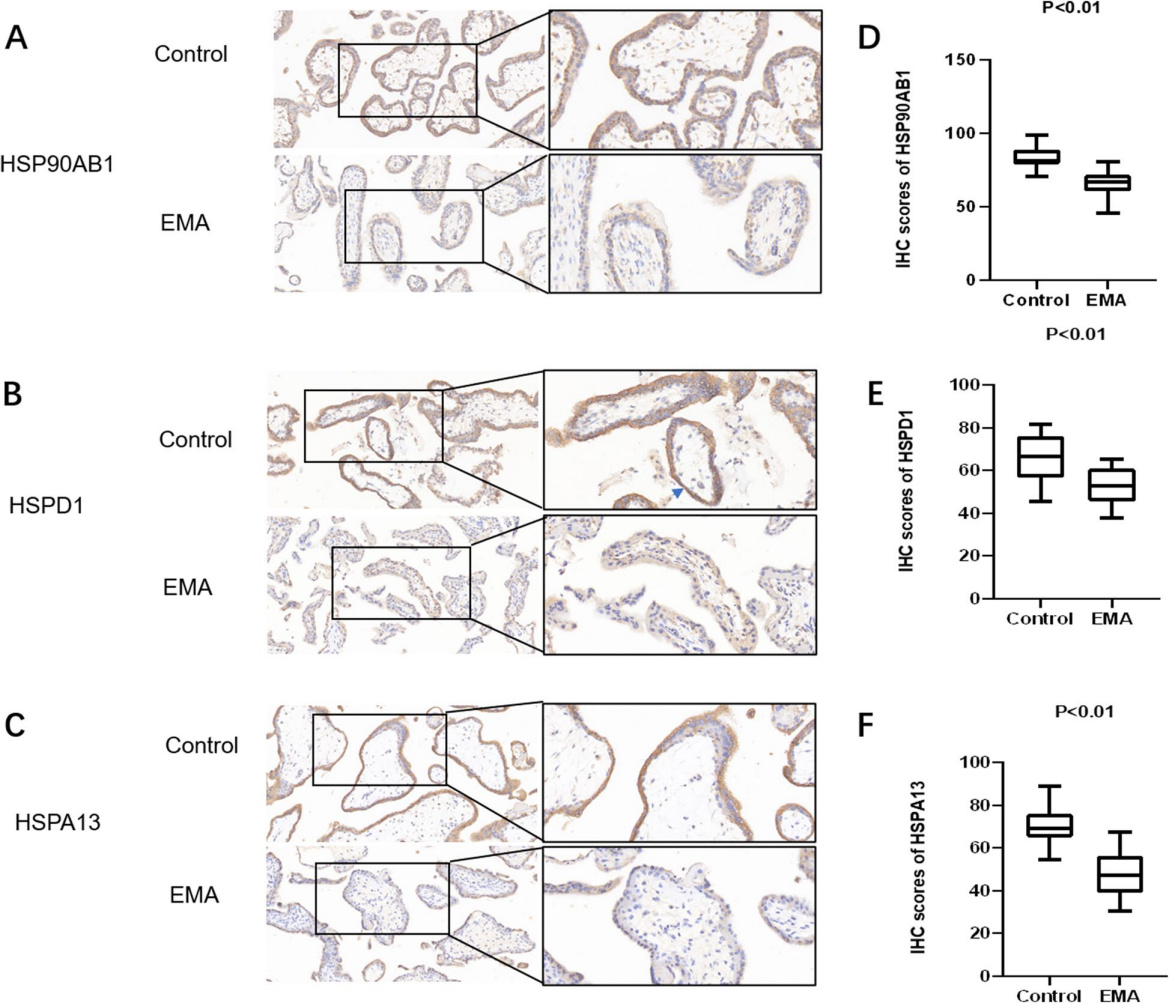
**A**,** B**,** C** Immunohistochemical staining for HSP90AB1, HSPD1, and HSPA13 observed in the cytoplasm of syncytiotrophoblast and cytotrophoblast cells (× 200, × 400). Villus from 22 patients with EMA showed lower; **D**-**F** Quantitative scoring results of IHC analyses w shown as box plots

Further analysis was conducted on the 3 candidate biomarkers HSP90AB1, HSPD1 and HSPA13 using IHC. Through staining, it was found that the levels of HSP90AB1, HSPD1 and HSPA13 in the villi of 22 cases of EMA were significantly lower than those in the control group (Fig. 3), which was consistent with the abundance trends detected by the PRM-based method.

## Discussion

PRM is a targeted mass spectrometry technique where a new-generation mass spectrometer with high resolution and high precision is used for ion monitoring of the target protein or peptide fragment to realize accurate quantification of the target protein/peptide fragment. In this study, a targeted quantitative proteomics method based on PRM was used to comprehensively analyze the differential abundance of heat shock proteins in EMA tissues. We observed some differences in the abundance of 16 target proteins in human villus and decidual tissues. Specifically, the levels of HSP90AB1, HSPD1 and HSPA13 were decreased in EMA villus tissues with significant differences, which was consistent with the results of Western blot and IHC verification. However, there were no statistically significant changes in these HSPs in decidual tissue, which may be related to the small size of our sample, and these results need to be further confirmed by expanding the sample size; on the other hand, these HSPs may only have changes in expression in villi. *Cui* et al. [13]. utilized proteomics to investigate factors related to early embryonic development and employed iTRAQ technology to compare protein profiles of serum samples from patients with normal pregnancies and cases of early recurrent spontaneous abortion (ERSA). Through this comparison, they identified 78 differentially expressed proteins. Furthermore, PRM technology was employed to validate three proteins—CD45, PSG1, and Prdx-2—which were found to be closely associated with miscarriage. There are some distinctions between their study and ours. First, we employed distinct clinical samples, focusing on human embryonic chorionic villus and maternal decidua tissues, while they utilized human serum samples. Moreover, we specifically investigated the differential abundance of HSP in the specimens, whereas they examined the differential abundance of all proteins in the specimens. In summary, our findings might provide important evidence for further research into the complex pathophysiological mechanism of EMA.

As a major isoform of the HSP90 family, HSP90AB1 (HSP90β) is usually constitutively expressed [14]. *Loones* et al. revealed that mouse embryos synthesize high levels of HSP90β during the preimplantation stage of development [15]. Further studies showed that mice with Hsp90β gene mutations failed to differentiate to form the placental labyrinth in the presence of normal Hsp90α abundance [16], suggesting that it plays a critical role in trophoblast differentiation. Moreover, invasion of the decidua by villous trophoblasts plays a crucial role in successful embryo implantation, and the mechanism of embryo implantation is similar to tumor invasion [17]. An increasing number of previous studies have demonstrated that upregulation of HSP90AB1 abundance can promote the invasion and metastasis of various cancers, such as gastric cancer, lung cancer, and colon cancer [18–20]. Therefore, it can be assumed that downregulation of HSP90AB1 abundance in aborted villus tissue may lead to reduced differentiation and invasion of trophoblast cells and affect embryo implantation. However, further studies are still needed to explore the mechanisms underlying this reduced abundance.

HSPD1 is constitutively expressed in mitochondria and cytoplasm and plays a key role in chaperoning, thermotolerance, apoptosis, cancer, immunology and embryonic development [21]. HSP60 expression promotes embryonic stem cell (ESC) differentiation and inhibits ESC apoptosis [22]. HSP60 was reported to promote progesterone synthesis [23] and regulate yolk sac erythropoiesis in mice [24]. Lipopolysaccharide-induced implantation failure may be related to significantly lower abundance of Hsp90, Hsp70, and Hsp60 [25]. Cumulatively, these findings suggest that HSP60 plays a distinct role during embryonic development and implantation. In our study, we verified that Hsp60 is obviously downregulated in villi of the EMA group compared with the control group. Similar to our study, using proteomics technology, Johnstone [26] et al*.* observed that HSP60 was downregulated in highly purified cytotrophoblasts from patients with preeclamptic placentas. Another study showed that the abundance of HSP27, HSP60, HSP70, and HSP90 in syncytiotrophoblasts (STs) and cytotrophoblasts around infarction region placentas with intrauterine fetal growth restriction was decreased due to lethal damage [27]. However, the abundance of HSP60 in peripheral blood was inconsistent with our data, which may be due to the elevation of HSP60 levels in plasma originating from sources other than the trophoblast layer [28]. Further research showed that HSP60 acts as a potent inhibitor of apoptosis by binding to proapoptotic regulators such as Bax, Bak, p21, p53, and survivin [29–31]. Gupta et al. observed that HSP60 could form a complex with Bax in the cytosol and inhibit its translocation to mitochondria, leading to the suppression of cell apoptosis [32]. Moreover, apoptosis is increased in EMA villi [33]. Therefore, we speculated that downregulation of HSP60 might promote the apoptosis of villous trophoblast cells and lead to EMA. Further studies will be conducted to verify this hypothesis.

HSPA13 is known as a member of the HSP70 family. To date, research on the biological function of HSPA13 in trophoblasts and its relationship with EMA is still relatively limited. Studies have shown that HSPA13 is overexpressed in colon and hepatocellular carcinoma tissues [34, 35]. Infiltrating gestational trophoblasts share many similarities with tumor cells in biological activity. Therefore, the downregulation of HSPA13 abundance in villus tissue may be critical in the development of EMA disease.

## Conclusion

In summary, through PRM-targeted quantitative proteomics, it was found in this study that HSP90AB1, HSPD1 and HSPA13 in villus tissue were candidate proteins with potential importance in EMA. Notably, this method enables more precise and efficient study of changes in protein profiles. However, this study is limited by the small sample size and lack of further functional studies on HSP90AB1, HSPD1 and HSPA13, so the specific mechanisms by which they are involved in the occurrence of EMA have not been clarified. In future studies, we will further investigate the specific functions of these three proteins and their roles in the pathological mechanisms of EMA and identify relevant therapeutic targets. In summary, their potential applications at the maternal–fetal interface would require larger and more in-depth studies.

### Supplementary Information

**Additional file 1:** **Fig. S1.** Western blot analysis to verify selected differentially expressed proteins HSP90AB1, HSPD1 and HSPA13; Candidate proteins were examined in triplicate and normalized to GAPDH levels for quantitative analysis; 1-6represent sample 1 to sample 6.

## Data Availability

The data supporting the findings of this study can be obtained from the corresponding author upon reasonable request.
