# Supplementary material for: PRM-based quantitative proteomics analysis of altered HSP abundance in villi and decidua of patients with early missed abortion
Source: Proteome Sci. 2023 Aug 16;21:12. doi: 10.1186/s12953-023-00213-w (PMC10429090; doi:10.1186/s12953-023-00213-w)
Supplement: Supplementary file 1 — Additional file 1: Fig. S1. Western blot analysis to verify selected differentially expressed proteins HSP90AB1, HSPD1 and HSPA13; Candidate proteins were examined in triplicate and normalized to GAPDH levels for quantitative analysis; 1-6represent sample 1 to sample 6. [file 12953_2023_213_MOESM1_ESM.docx]

**PRM-based quantitative proteomics analysis of altered HSPs expression in villi and decidua of**

**early missed abortion patients**

**Supplementary material** **Fig.1** Western blot analysis to verify selected differentially expressed proteins HSP90AB1, HSPD1 and HSPA13; Candidate proteins were examined in triplicate and normalized to GAPDH levels for quantitative analysis；1-6represent sample 1 to sample 6


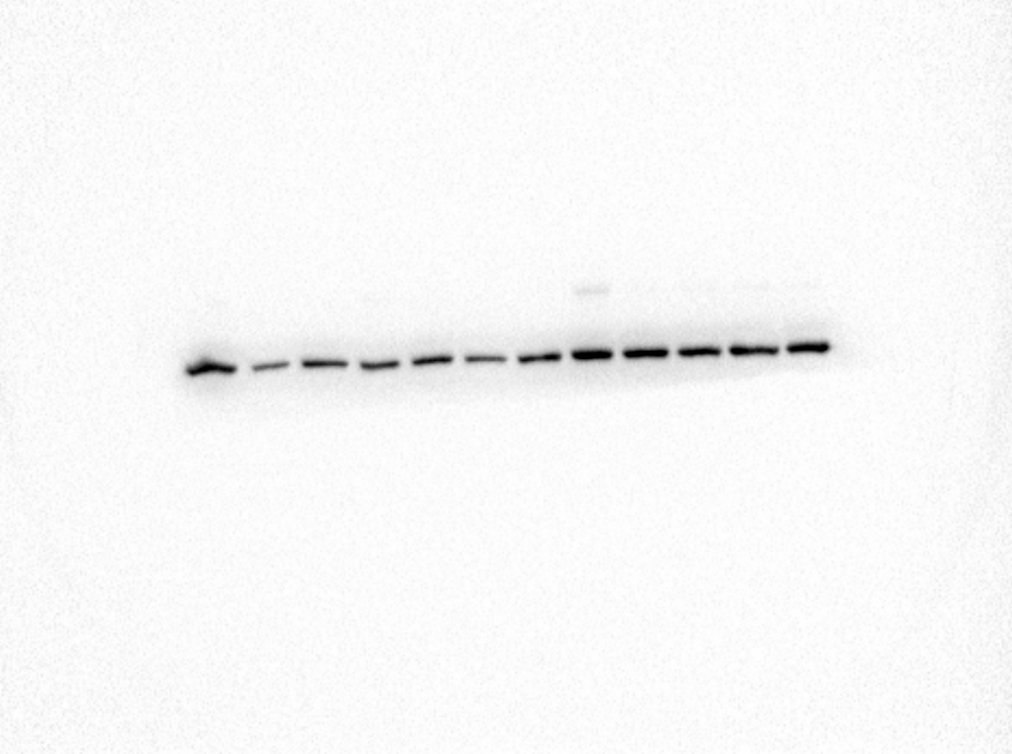


HSP90AB1 90kDa

1 2 3 4 5 6 1 2 3 4 5 6

EMA group Control group


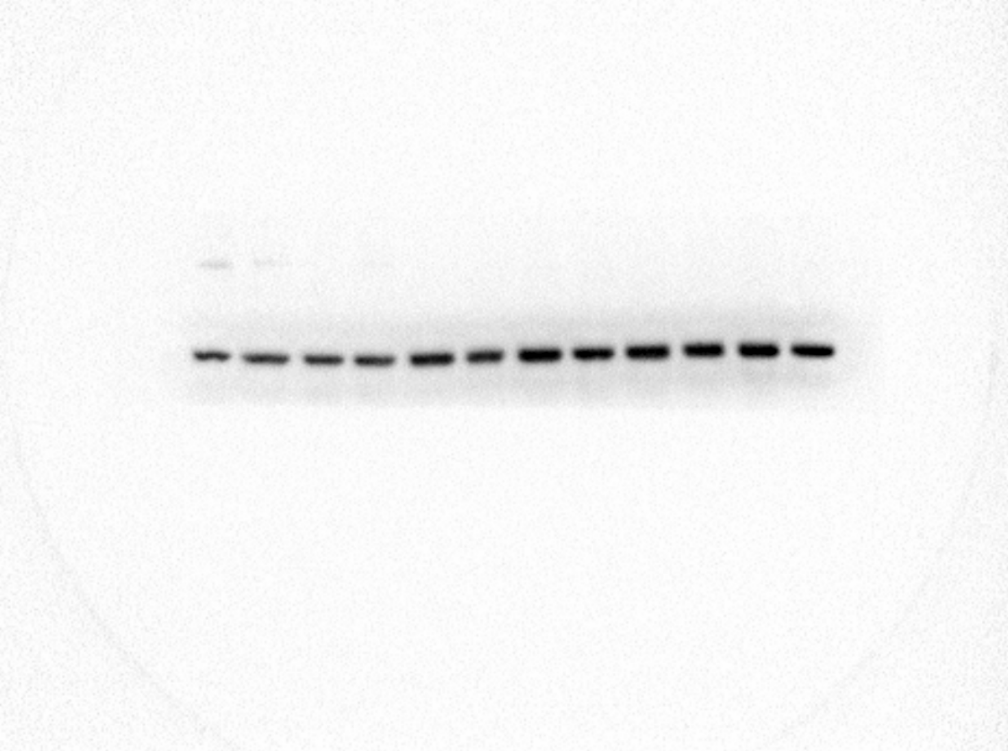


HSPD1 60 kDa

1 2 3 4 5 6 1 2 3 4 5 6

EMA group Control group


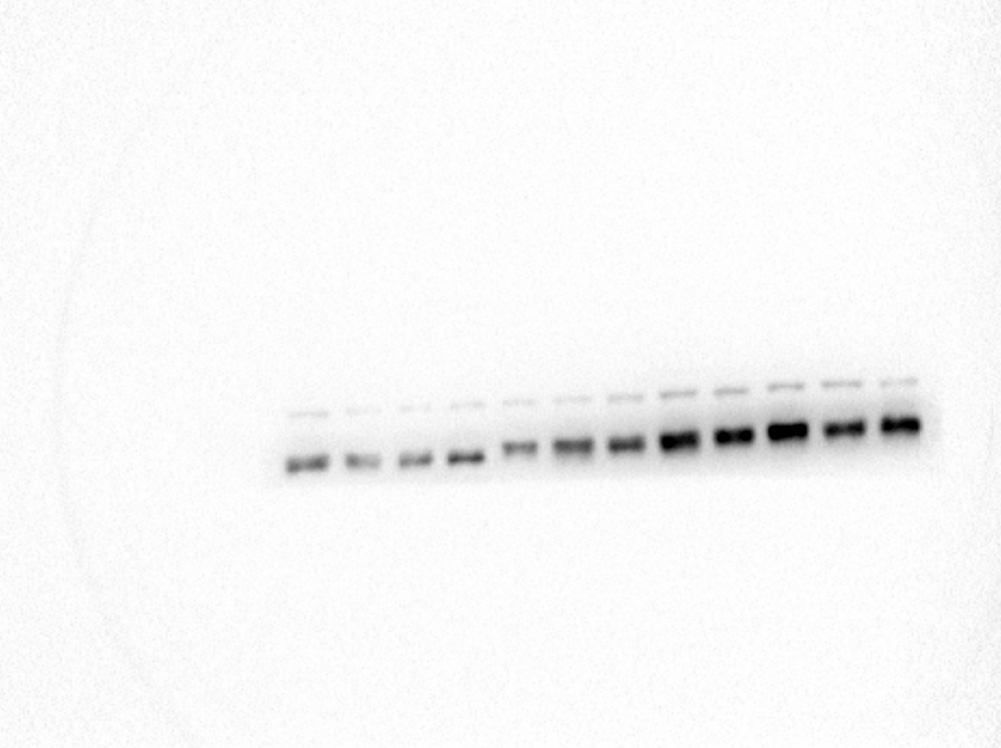


HSPA13 52kDa

1 2 3 4 5 6 1 2 3 4 5 6

EMA group Control group


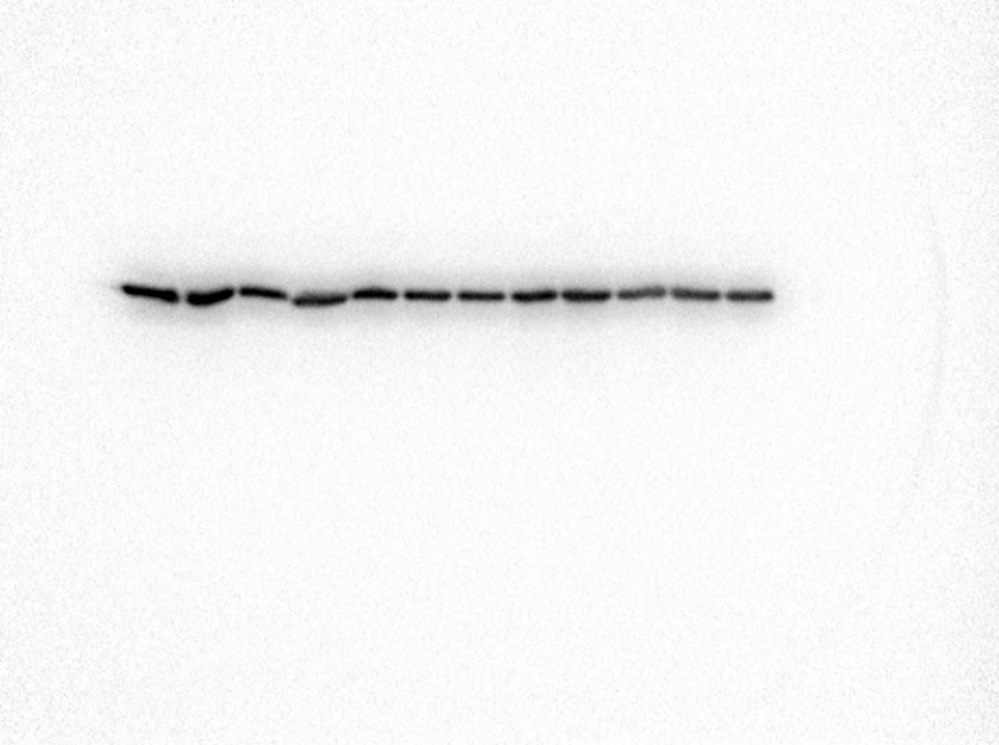


GAPDH 35.9kDa

1 2 3 4 5 6 1 2 3 4 5 6

EMA group Control group
